# Effects of acids, pepsin, bile acids, and trypsin on laryngopharyngeal reflux diseases: physiopathology and therapeutic targets

**DOI:** 10.1007/s00405-021-07201-w

**Published:** 2021-12-03

**Authors:** Yading Li, Gaofan Xu, Bingduo Zhou, Yishuang Tang, Xiaowen Liu, Yue Wu, Yi Wang, Jing Kong, Tingting Xu, Cong He, Shengliang Zhu, Xiaosu Wang, Jianning Zhang

**Affiliations:** Yueyang Hospital of Integrated Traditional Chinese and Western Medicine, Shanghai University of Traditional Chinese Medicine, 110 Ganhe Road, Hongkou District, Shanghai, 200437 China

**Keywords:** Laryngopharyngeal reflux, Reflux substance, Mechanistic studies, Precancerous conditions, Therapeutic target

## Abstract

**Purpose:**

Laryngopharyngeal reflux disease (LPRD) is a general term for the reflux of gastroduodenal contents into the laryngopharynx, oropharynx and even the nasopharynx, causing a series of symptoms and signs. Currently, little is known regarding the physiopathology of LPRD, and proton pump inhibitors (PPIs) are the drugs of choice for treatment. Although acid reflux plays a critical role in LPRD, PPIs fail to relieve symptoms in up to 40% of patients with LPRD. The influence of other reflux substances on LPRD, including pepsin, bile acid, and trypsin, has received increasing attention. Clarification of the substances involved in LPRD is the basis for LPRD treatment.

**Methods:**

A review of the effects of acids, pepsin, bile acids, and trypsin on laryngopharyngeal reflux diseases was conducted in PubMed.

**Results:**

Different reflux substances have different effects on LPRD, which will cause various symptoms, inflammatory diseases and neoplastic diseases of the laryngopharynx. For LPRD caused by different reflux substances, 24-h multichannel intraluminal impedance combined with pH-metry (MII-pH), salivary pepsin, bile acid and other tests should be established so that different drugs and treatment courses can be used to provide patients with more personalized treatment plans.

**Conclusion:**

This article summarizes the research progress of different reflux substances on the pathogenesis, detection index and treatment of LPRD and lays a theoretical foundation to develop target drugs and clinical diagnosis and treatment.

## Introduction

Laryngopharyngeal reflux disease (LPRD) is a general term for the reflux of gastroduodenal contents into the laryngopharynx, oropharynx and even nasopharynx, causing a series of symptoms and signs [1]. Although LPRD was first proposed by the American Academy of Otolaryngology-Head and Neck Surgery in 2002 [2], its pathogenesis and treatment remain controversial. Most scholars believe that LPRD is a manifestation of extraesophageal symptoms of gastroesophageal reflux disease (GERD) [3], and the pathogenesis is essentially the same as that of GERD. However, an indirect stimulation mechanism of the vagal reflex exists in LPRD, and the presence of a proton pump (H+/K+-ATPase) in the larynx may induce acid production and mucosal damage [4]. According to the 24-h multichannel intraluminal impedance combined with pH-metry (MII-pH) and definition consensus, the types of reflux can be classified as acid reflux, weak acid reflux, alkaline reflux, nonacid reflux, and mixed reflux [5, 6]. Reflux substances comprise different levels of hydrochloric acid, pepsin, bile, and trypsin. These substances are the main components that cause laryngopharyngeal mucosal damage [7]. Presently, proton pump inhibitors (PPIs) are the first choice for LPRD treatment, but PPIs fail to relieve symptoms in up to 40% of patients with LPRD [8]. Substantial clinical evidence is lacking regarding the effectiveness of PPIs on nonacidic LPRD [9, 10]; thus, more studies have focused on drug development for nonacidic LPRD. In an international research survey, 21.1% of otolaryngologists estimated that the prevalence rates of nonacidic LPRD and mixed LPRD were 25.4% and 35.5% of all LPRD patients, respectively [11]. Given the complexity of the symptoms and mechanisms of LPRD and doubtful efficacy of PPIs, it is important to study the effects of different reflux substances on LPRD.

## Mechanistic effects of reflux material on LPRD

### Hydrochloric acid

Hydrochloric acid is a major determinant of esophageal irritation and reflux symptoms [12]. The laryngeal mucosa is more sensitive to acid stimulation than the esophagus [13]. Even small amounts of acid can cause severe damage to the laryngeal mucosa. Exploring how the larynx is exposed to acidic conditions is the key to determining the pathogenesis of LPRD.

The acid in the gastric juice or ectopic gastric mucosa of the upper esophagus causes reflux due to esophageal barrier dysfunction and contacts the laryngopharyngeal tissue, causing damage to laryngopharyngeal mucosal epithelial cells and inflammation. The mechanism may be related to two aspects. (1) Carbonic anhydrase III (CA III) can actively secrete bicarbonate and adjust the pH value to address acid reflux. The lack of CA III in the laryngeal tissue of some patients with LPRD leads to an imbalance in pH regulation [14]. (2) E-cadherin is a transmembrane glycoprotein in epithelial tissue that affects intercellular adhesive junctions. It forms a permeable barrier in the epithelial cells of the pharynx and nose, preventing the diffusion of most solutes and maintaining tight junctions between the cells. Acid reflux can reduce the expression of E-cadherin and cause increased intercellular permeability, thereby damaging pharyngeal and nasal mucosal cells [15, 16].

On the other hand, H+/K+-ATPase is a key enzyme involved in acid secretion. H+/K+-ATPase is mainly distributed in the surface layer of gastric parietal cells and secretes hydrochloric acid into the gastric lumen through the exchange of H+ and K+. Altman et al. [17, 18] demonstrated that H+/K+-ATPase is present in serous cells and ducts of submucosal glands in the human larynx. Although its concentration is much lower than that in the stomach, this may be another cause of acid exposure [4]. The expression level of H+/K+-ATPase is higher in laryngeal cancer tissues than in normal laryngeal tissues [19]. High expression of H+/K+-ATPase leads to abnormal acid secretion, causing local inflammation, destruction of mitochondria and cell carcinogenesis [20]. In addition, H+-ATPase channels can function as an auxiliary or secondary acid secretion pathway even under conditions of K+ depletion or pharmacological inhibition of the proton pump [21]. In summary, hydrochloric acid acts on CA III, E-cadherin and laryngeal H+/K+-ATPase to cause laryngopharyngeal damage. The physiological role of laryngeal H+/K+-ATPase will be the focus of future research.

### Pepsin

The abnormal secretion and activation of pepsin are crucial to the pathogenesis of LPRD. Pepsin is converted from pepsinogen produced by gastric chief cells and is a major factor causing proteolysis and cell damage. Pepsin is undetectable in the laryngeal mucosa of healthy individuals [22]. Pepsin remains active at pH 2.0–6.0 [23]. At pH 5.5 and 6.0, it has approximately 30% and 10% activity, respectively. Under neutral conditions, it remains stable, although it is inactive [24]. Bulmer et al. [13] found that the laryngeal mucosa is essentially resistant to injury at pH 4.0, but when pepsin is present, it is extremely vulnerable.

The mechanism of laryngopharyngeal mucosal injury caused by pepsin mainly includes the following. (1) Pepsin can downregulate E-cadherin and reduce cell adhesion, leading to the release and accumulation of β-catenin from the cell membrane to the cytoplasm, thereby increasing the possibility of tumor cell infiltration and metastasis [25, 26]. Pepsin can also combine with CXC chemokine receptor 2 (CXCR 2) by inducing the secretion of interleukin (IL)-8 and ultimately altering the levels of E-cadherin/β-catenin [27]. (2) Pepsin is reactivated by re-exposure to an acidic environment or transport to a cell environment with a low pH. It enters cells through endocytosis and is stored in vesicles or transported to other complex organelles (such as the Golgi apparatus), causing mitochondrial damage and promoting the expression of many tumor-related genes in a cell environment with a low pH [28]. (3) Pepsin reduces the expression of CA III and attenuates the neutralization of CA III on acid [29]. (4) It is involved in the stress response mediated by squamous epithelium stress proteins (Sep), leading to impaired laryngopharyngeal mucosal cell function [30]. Sep not only show protective effects on cell stress but also participate in the repair or removal of damaged peptides. Pepsin leads to a disruption of the laryngeal barrier by reducing Sep70 and Sep53 levels in a low pH environment [31]. (5) Pepsin increases the levels of mucin 5AC mRNA and glycoproteins in airway epithelial cells through matrix metalloproteinase (MMP)-9 and nuclear factor kappa-B (NF-κB) pathways, promoting the secretion of airway mucus hypersecretion and causing airway inflammation [32]. In addition, Doukas et al. [33] showed that pepsin, at a neutral pH of 7.0, is more likely to cause NF-κB and signal transducer and activator of transcription factor 3 (STAT3) activation and upregulation than a weakly acidic pH of 5.0–6.0, which upregulates growth factor receptor (EGFR), AKT1, mammalian target of rapamycin (mTOR), IL-1β, tumor necrosis factor-α (TNF-α), RelA/p65, B cell lymphoma 2 (BCL-2) and IL-6. In another study performed by Niu et al. [34], pepsin induced the activation of NF-κB, tumor necrosis factor-related apoptosis inducing ligand (TRAIL) and NOTCH signaling, representing major mediators of cell proliferation, differentiation and apoptosis. (6) Pepsin increases the expression of 8-hydroxy-2'-deoxyguanosine (8-OHdG) and p-H2AX, which promotes DNA oxidative damage and double-strand breaks (DSB) [35]. (7) Pepsin causes cell damage and increases cancer risk through the endocytosis of lipoprotein receptor-related 1 (LRP1)/alpha-2 macroglobulin (α-2M) [36]. In essence, pepsin causes laryngopharyngeal damage through CA III, IL-8, Sep, E-cadherin, NF-κB and other channels in different pH environments. How to prevent pepsin from being activated is the key to treatment.

Pepsin not only damages the laryngopharyngeal mucosa but also induces chronic inflammation of the surrounding tissues to cause vocal fold polyp [37], tonsillar hypertrophy [38, 39], otitis media [40], recurrent respiratory papillomatosis [22, 41], laryngopharyngeal tumors [34, 36] and other diseases.

### Bile

Bile reflux is a major cause of inflammatory damage and cellular carcinoma of the laryngopharynx and is associated with laryngotracheal stenosis, tracheal fibrosis and laryngotracheal malignancy [42]. Bile is secreted by the liver. Bile acid is the main component of bile, which maintains fat digestion and absorption, regulates inflammation, and affects the intestinal flora [43]. Studies have shown that bile acid is an independent risk factor for laryngeal cancer. The prevalence of LPRD in patients with laryngeal cancer is as high as 67% [44]. The bile acid level correlates positively with symptom severity and the risk of laryngeal cancer in patients with LPRD [45]. De Corso et al. [46] found that bile reflux after gastrectomy increased the risk of laryngeal cancer by 10 times, and the incidence of laryngeal leukoplakia was also higher.

The main mechanisms by which bile acid causes pharyngeal inflammation and cell carcinogenesis include the following. (1) Bile acid induces the epithelial–mesenchymal transition (EMT) in cells. EMT refers to the transformation of epithelial cells into mesenchymal cells, allowing them to migrate and invade. Bile acid induces transforming growth factor-β1 (TGF-β1) through EMT channels, causing a decrease in E-cadherin and an increase in MMP-9 and fibronectin, leading to laryngotracheal scar formation, airway remodeling and tumor growth [42]. (2) NF-κB activation alters the expression of tumor transformation-related molecules and produces selective carcinogenic effects on the hypopharyngeal mucosa. Sasaki et al. [47] found that compared with hypopharyngeal squamous cell carcinoma (HSCC) specimens without bile reflux, NF-κB was significantly activated and altered the IL-6, IL-1β, EGFR, STAT3, TNF-α, BCL-2, RelA/p65, cREL, ΔNp63, Wnt5a and microRNA expression levels in HSCC specimens with typical bile reflux. At the same time, the authors confirmed through animal and in vitro experiments that the combination of bile acid and hydrochloric acid induces NF-κB activation, changes the expression of tumor transformation-related molecules and early histopathology, and leads to uncontrolled changes in tumor suppressor microRNAs (miR-21, miR-155, miR-192, and miR-375) [48–51]. They have been further confirmed to increase the Trp53 protein, accompanied by DNA/RNA oxidative damage and increased positivity for γH2AX, a marker of DSB [52]. However, even at weakly acidic pH (5.5–6.0), bile acids can promote DNA/RNA damage, NF-κB activation, and precancerous lesions of the mRNA and miRNA phenotypes [53]. In short, bile acids mainly act on EMT and NF-κB channels to induce abnormal expression of tumor factors.

### Trypsin

Trypsin is secreted by pancreatic cells in the form of zymogen. It is the most effective activator of proteinase-activated receptor-2 (PAR-2). PAR-2 is involved in intestinal inflammation and the neurogenic inflammatory epithelial response. It is expressed in esophageal epithelial cells [54], odontoblasts [55], sinus epithelial ciliated cells [56] and others.

PAR-2 activation by trypsin affects the regulation of the lower esophageal sphincter (LES). LES dysfunction underlies the pathogenesis of LPRD. The LES includes circular smooth muscle (CSM) and longitudinal smooth muscle (LSM), and activated PAR-2 functions mainly via bidirectional (systolic and diastolic) mechanisms in CSM. Trypsin stimulation of PAR-2 activates transient receptor potential vanilloid type (TRPV) 1 of capsaicin-sensitive sensory neurons in the CSM. TRPV1 releases substance P (SP), which activates natural killer receptors (NKR) 1/2 to induce contraction in CSM [57]. Tanaka et al. [58] concluded that PAR-2 induces the activation of Rho-associated protein kinase (ROK), p38 mitogen-activated protein kinase (p38 MAPK) and extracellular signal-regulated protein kinase (ERK) 1/2. ROK is involved in the contractile function of CSM, whereas ROK, p38 MAPK, ERK1/2, and membrane hyperpolarization are involved in relaxation.

Trypsin activates PAR-2 to induce the secretion of IL-8 and TRPV, causing epithelial barrier dysfunction, which mainly occurs in the basal layer of the squamous epithelium of the esophagus [59] and larynx [60]. IL-8 is a neutrophil chemotactic factor involved in the inflammatory response. TRPV has a heat-sensitive, mechanosensitive role and includes subtypes such as TRPV1 and TRPV4. TRPV1 participates in the processes of inflammation and immune activation, aggravates heartburn and pain symptoms in LPRD, and damages the epithelial mucosa [61]. TRPV4 is dependent on calcium inward flow and facilitates ATP cytosolic release, which is involved in esophageal mechanical and thermal stimulation and impairs esophageal barrier function [62]. Obviously, the main target of trypsin is PAR-2 and TRPV, causing LES abnormalities and heat sensitivity, among other effects.

Other studies have shown that trypsin increases pulmonary aspiration injury. In addition, it can survive in the oral cavity, degrading eroded dentin and causing increased tissue loss [63].

**Fig. 1:**
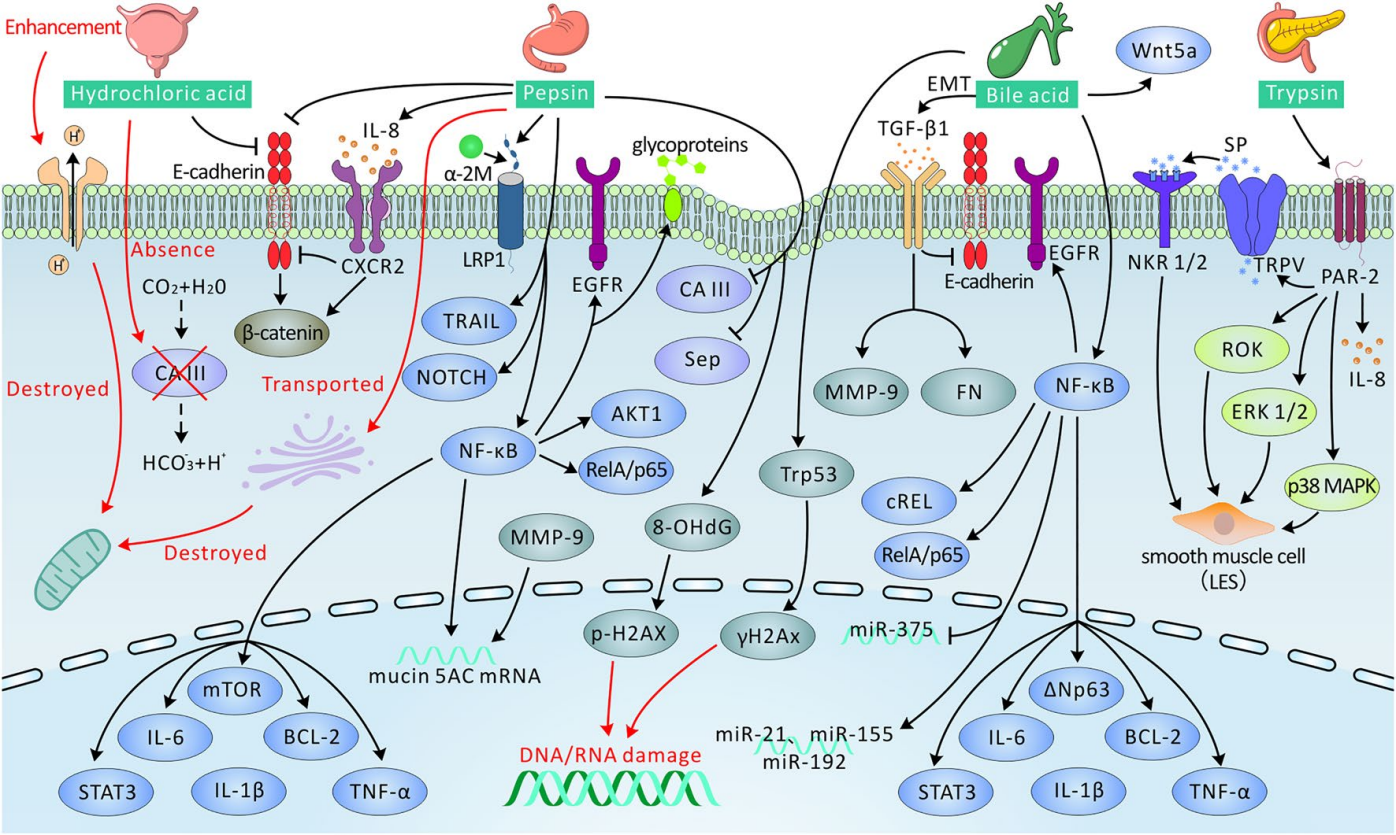
Potential mechanisms of reflux material in LPRD patients (details are provided in the text). Arrows terminating with → represent activation, while those terminating with ┴ represent inhibition/deterioration. *CA III* carbonic anhydrase III, *IL* interleukin, *CXCR 2* CXC chemokine receptor 2, *Sep* squamous epithelium stress proteins, *NF-κB* nuclear factor kappa-B, *STAT3* signal transducer and activator of transcription factor 3, *EGFR* epidermal growth factor receptor, *mTOR* mammalian target of rapamycin, *TNF-α* tumor necrosis factor-α, *BCL-2* B cell lymphoma 2, *TRAIL* tumor necrosis factor-related apoptosis inducing ligand, *8-OHdG* 8-hydroxy-2'-deoxyguanosine, *LRP1* lipoprotein receptor-related 1, *α-2M* alpha-2 macroglobulin, *EMT* epithelial–mesenchymal transition, *TGF-β1* transforming growth factor-β1, *MMP-9* matrix metalloproteinase-9, *miR* microRNA, *PAR-2* proteinase-activated receptor-2, *TRPV* transient receptor potential vanilloid type, *SP* substance P, *ROK* Rho-associated protein kinase, *NKR 1/2* natural killer receptors 1/2, *p38 MAPK* p38 mitogen-activated protein kinase, *ERK 1/2* extracellular signal-regulated protein kinase 1/2, *LES* lower esophageal sphincter

Overall, the physiopathology of LPRD disease is complex and caused by acids, pepsin, bile acids, and trypsin (Fig. 1; Table 1).

**Table 1:** The functional roles of cytokines and cellular receptors in LPRD patients

Reflux material	Cytokines/cellular receptors	Function	References
Hydrochloric acid	CA III ↓	Secrete bicarbonate and adjust pH	[14]
	E-cadherin ↓	Maintain tight junctions between cells	[15, 16]
	H+/K+-ATPase, H+-ATPase ↑	Secrete acid	[4, 17–21]
Pepsin	E-cadherin ↓	Maintain tight junctions between cells	[25, 26]
	IL-8, CXCR 2 ↑	Change the levels of E-cadherin/β-catenin	[27]
	CA III ↓	Secrete bicarbonate and adjust pH	[29]
	Sep70, Sep53 ↓	Protect cells from stress and repair or remove damaged peptides	[30, 31]
	Mucin 5AC mRNA, glycoproteins ↑	Promotes secretion of airway mucus hypersecretion and causes airway inflammation	[32]
	NF-κB, STAT3, EGFR, AKT1, mTOR, IL-1β, TNF-α, RelA/p65, BCL-2, IL-6, TRAIL, NOTCH signaling ↑	Lead to cell proliferation, differentiation and apoptosis	[33, 34]
	8-OHdG, p-H2AX ↑	Promote DNA oxidative damage and double-strand breaks	[35]
	LRP1, α-2M	Causes cell damage and increased cancer risk	[36]
Bile	EMT, TGF-β1, MMP-9, fibronectin ↑; E-cadherin ↓	Cause cell migration and invasion	[42]
	NF-κB, IL-6, IL-1β, EGFR, STAT3, TNF-α, BCL-2, RelA/p65, cREL, ΔNp63, Wnt5a, miR-21, miR-155, miR-192 ↑; miR-375 ↓	Cause throat cancer	[47–51]
	Trp53, γH2Ax ↑	Promote DNA/RNA damage and precancerous lesions	[52, 53]
Trypsin	PAR-2, TRPV1, SP, ROK, NKR 1/2, p38 MAPK, ERK1/2 ↑	Involved in the contraction and diastole of CSM	[57, 58]
	PAR-2, TRPV, IL-8 ↑	Cause inflammation and immune activation	[59–62]

Arrows terminating with ↑ represent activation, while those terminating with ↓ represent inhibition/deterioration

*CA III* carbonic anhydrase III, *IL* interleukin, *CXCR 2* CXC chemokine receptor 2, *Sep* squamous epithelium stress proteins, *NF-κB* nuclear factor kappa-B, *STAT3* signal transducer and activator of transcription factor 3, *EGFR* epidermal growth factor receptor, *mTOR* mammalian target of rapamycin, *TNF-α* tumor necrosis factor-α, *BCL-2* B cell lymphoma 2, *TRAIL* tumor necrosis factor-related apoptosis inducing ligand, *8-OHdG* 8-hydroxy-2'-deoxyguanosine, *LRP1* lipoprotein receptor-related 1, *α-2M* alpha-2 macroglobulin, *EMT* epithelial–mesenchymal transition, *TGF-β1* transforming growth factor-β1, *MMP-9* matrix metalloproteinase-9, *miR* microRNA, *PAR-2* proteinase-activated receptor-2, *TRPV* transient receptor potential vanilloid type, *SP* substance P, *ROK* Rho-associated protein kinase, *NKR 1/2* natural killer receptors 1/2, *p38 MAPK* p38 mitogen-activated protein kinase, *ERK 1/2* extracellular signal-regulated protein kinase 1/2, *CSM* circular smooth muscle

## Interaction between different reflux materials

When many types of reflux substances are found in reflux fluid, the damage to the hypopharyngeal mucosa is more severe. Lee et al. [5] compared nonacidic reflux and mixed reflux in LPRD and found that patients with mixed reflux had more severe cough, globus sensation and distal reflux.

Under the action of hydrochloric acid, bile acid is protonated, penetrates and dissolves the cell membrane, enhancing its cytotoxic effect [7]. At the same time, the proteolytic process of pepsin can proceed, causing neutrophils to disrupt the integrity of the epithelial barrier [64]. Roh et al. [65] found that pepsin and bile acid damage the subglottic tissue more severely under acidic conditions (pH 1–2). Doukas et al. [66] found that strong acidic conditions (pH ≤ 4.0) enhance the carcinogenic effects of bile acids on hypopharyngeal cells.

Previous studies have suggested that bile acids inhibit pepsin activity [67]. However, Ali et al. [24] concluded that bile acids do not attenuate pepsin activity and that the combination of the two enhances the damaging effect under nonacidic conditions. Presently, the mechanism of the interaction between bile acids and pepsin is unclear.

## Research and application of reflux substances as detection indicators

Presently, 24-h MII-pH is considered the method of choice for diagnosing LPRD and can identify liquid, gaseous or mixed forms and detect both acid and nonacid reflux [6, 68]. However, it is invasive, time consuming and expensive, limiting its widespread use in clinical practice. The methods used to detect pepsin, bile acid, Sep70 and MMP are simple and highly feasible. However, their detection standards, sensitivity and specificity are not the same in different research centers; thus, they remain unsuitable for the clinical diagnosis of LPRD [69]. The positive threshold of pepsin and collection time of saliva specimens remain inconclusive [70]. Na et al. [71] concluded that the best time for saliva collection is the just awake state of LPRD patients. De Corso et al. [45] concluded that bile acid is most suitable to diagnose LPRD, with a sensitivity of 86% and a positive predictive value of 80.7%. Salivary bile acid > 1 µmol/L is a reliable indicator to evaluate the severity of LPRD. Hoppo et al. [72] suggested that Sep70 is a “protective” indicator, and its absence indicates hypopharyngeal cell damage. The Sep70/pepsin ratio may be a more reliable indicator to reflect the actual damage of LPRD, with a sensitivity as high as 91%. However, its specificity is very low, and its clinical application value warrants further study. Im et al. [73] found that the sensitivity and specificity of MMP-7 as a detection marker were 71.43% and 79.75%, respectively. When MMP-7 and pepsin were combined for detection, the sensitivity and specificity increased to 80% and 82.28%, respectively.

## Current status of LPRD treatment for different reflux substances

### Gastric acid-inhibiting drugs

PPIs are the drugs of choice to treat LPRD and are suitable for LPRD patients with typical GERD symptoms. Depending on severity, the main therapeutic scheme consisted of twice daily PPIs, once daily high-dose PPIs or once daily PPIs for a duration ranged from 1 to 6 months [74, 75]. The most therapeutic scheme for American-Broncho-Esophagological Association (ABEA) members [76] and Brazilian otolaryngologists [77] is twice daily PPIs for 2–3 months. However, European general practitioners prefer once daily PPIs [78]. Park et al. [79] found that twice daily PPIs are more effective than once daily PPIs in achieving clinical symptom response in suspected LPR, with a treatment duration of at least 2 months. However, their efficacy remains controversial for the following reasons. (1) The long-term use of PPIs increases the risk of gastric tumors, acute nephritis, etc. [80] (2) The American Academy of Gastroenterology does not recommend using PPIs diagnostic tests for patients with atypical reflux [74]. For patients with suspected LPRD, using PPIs diagnostic tests will increase economic costs [9]. (3) Current meta-analyses have shown that PPIs are not superior to placebo in treating suspected LPRD [81, 82]. (4) The failure of high-dose PPIs treatment does not rule out LPRD, and 24-h MII-pH is still required [83]. (5) Patients with acid reflux in whom PPIs are ineffective can try switching to potassium-competitive acid blockers, which have a rapid onset of action and long-lasting acid suppression [84].

Patients who do not respond to empiric PPIs therapy (twice daily for 2–3 months) should be monitored for reflux [85]. If acid reflux is clearly present, the PPIs regimen can be optimized, including adjusting the administration time and dose, changing the PPI, and paying attention to nocturnal acid breakthrough [74]. When increasing the dose and prolonging the treatment time, the potential adverse risks should be carefully considered [86].

### Alginate

Following exposure to gastric acid, alginate forms a viscous, gelatinous neutral layer or barrier on top of the gastric contents to produce a mechanical barrier against the reflux of gastric acid or nonacidic substances, thereby reducing or preventing contact between gastric contents and the esophageal or laryngopharyngeal mucosa. In addition, alginate has a significant inhibitory effect on pepsin [87]. Wilkie et al. [88] confirmed that alginate alone effectively relieved LPRD symptoms, but no additional benefit was found when used in combination with high-dose PPIs. Darwish et al. [89] found that alginate raft-forming formulations containing nizatidine rapidly relieved burning symptoms.

### Enzyme inhibitors or receptor antagonists

Considering the different impact mechanisms of various reflux substances on LPRD, various enzyme inhibitors or receptor antagonists, including pepsin inhibitors or receptor antagonists, trypsin inhibitors, matrix metalloproteinase inhibitors (MMPIs), NF-κB antagonists, PAR-2 antagonists, and TRPV1 antagonists, have become new therapeutic targets. Johnston et al. [90] studied a drug that targets pepsin using the following mechanisms: (1) it irreversibly inactivates the enzyme to prevent its reactivation in cells at a low pH; and (2) receptor antagonists prevent pepsin uptake through receptor-mediated endocytosis. MMP is an enzyme that destroys E-cadherin. Kim et al. [16] found that MMPI blocks the cleavage of E-cadherin by MMP, reduces changes in cell permeability, and maintains intercellular communication, restoring the mucosal epithelial barrier function of patients with LPRD. Clinical studies have reported [91] that trypsin inhibitors improve the symptoms of reflux esophagitis after distal gastrectomy. Vageli et al. [50, 92] found that an NF-κB antagonist (BAY 11-7082) reduced the expression of NF-κB and related oncogenes induced by bile acids. Souza [93] proposed that the unresolved symptoms of heartburn after PPIs treatment may be mediated by PAR-2. PAR-2 and TRPV1 antagonists are expected to serve as targeted drugs to improve heartburn and pain caused by LPRD. Quilty et al. [94] found that p38 inhibitors, MEK inhibitors, PKC inhibitors and methyl-β-cyclodextrin through MAPK signaling pathways, particularly via p38 and Erk1/2, reduce IL-6 or IL-8 secretion, decreasing esophageal inflammation and treating GERD.

## Prospect

For LPRD caused by different reflux substances, 24-h MII-pH, salivary pepsin, bile acid and other tests should be established so that different drugs and treatment courses can be used to provide patients with more personalized treatment plans. For patients with nonacid reflux or refractory LPRD, PPI medication indications and discontinuation plans require multidisciplinary collaborative evaluations such as gastroenterology and otolaryngology. The poor efficacy of PPIs and precancerous lesions in the laryngopharynx indicates that the molecular mechanism of nonacidic components on laryngopharyngeal mucosal injury requires further study to reduce the recurrence rate of LPRD and incidence of malignant tumors. In addition, many clinical prospective studies are required to evaluate whether biomarkers such as pepsin and bile acids are reliable as diagnostic and prognostic indicators of LPRD to improve the current status of LPRD treatment. Many prospective studies are needed to evaluate whether the prognosis of LPRD can be improved using pepsin and bile acid as biomarkers.

## Data Availability

Not applicable.

## References

[1] Lechien JR, Saussez S, Karkos PD (2018). Laryngopharyngeal reflux disease: clinical presentation, diagnosis and therapeutic challenges in 2018. Curr Opin Otolaryngol Head Neck Surg.

[2] Koufman JA, Aviv JE, Casiano RR, Shaw GY (2002). Laryngopharyngeal reflux: position statement of the committee on speech, voice, and swallowing disorders of the American Academy of Otolaryngology-Head and Neck Surgery. Otolaryngol Head Neck Surg.

[3] Katzka DA, Kahrilas PJ (2020). Advances in the diagnosis and management of gastroesophageal reflux disease. BMJ.

[4] Becker V, Drabner R, Graf S, Schlag C, Nennstiel S, Buchberger AM, Schmid RM, Saur D, Bajbouj M (2015). New aspects in the pathomechanism and diagnosis of the laryngopharyngeal reflux-clinical impact of laryngeal proton pumps and pharyngeal pH metry in extraesophageal gastroesophageal reflux disease. World J Gastroenterol.

[5] Lee JS, Jung AR, Park JM, Park MJ, Lee YC, Eun Y (2018). Comparison of characteristics according to reflux type in patients with laryngopharyngeal reflux. Clin Exp Otorhinolaryngol.

[6] Sifrim D, Castell D, Dent J (2004). Gastro-oesophageal reflux monitoring: review and consensus report on detection and definitions of acid, non-acid, and gas reflux. Gut.

[7] Sharma P, Yadlapati R (2020). Pathophysiology and treatment options for gastroesophageal reflux disease: looking beyond acid. Ann N Y Acad Sci.

[8] Lechien JR, Akst LM, Hamdan AL, Schindler A, Karkos PD, Barillari MR, Calvo-Henriquez C, Crevier-Buchman L, Finck C, Eun Y (2019). Evaluation and management of laryngopharyngeal reflux disease: state of the art review. Otolaryngol Head Neck Surg.

[9] Carroll TL, Werner A, Nahikian K, Dezube A, Roth DF (2017). Rethinking the laryngopharyngeal reflux treatment algorithm: evaluating an alternate empiric dosing regimen and considering up-front, pH-impedance, and manometry testing to minimize cost in treating suspect laryngopharyngeal reflux disease. Laryngoscope.

[10] Lechien JR, Bock JM, Carroll TL, Akst LM (2020). Is empirical treatment a reasonable strategy for laryngopharyngeal reflux? A contemporary review. Clin Otolaryngol.

[11] Lechien JR, Allen JE, Barillari MR, Karkos PD, Jia H, Ceccon FP, Imamura R, Metwaly O, Chiesa-Estomba CM, Bock JM (2020). Management of laryngopharyngeal reflux around the world: an international study. Laryngoscope.

[12] Tack J, Pandolfino JE (2018). Pathophysiology of gastroesophageal reflux disease. Gastroenterology.

[13] Bulmer DM, Ali MS, Brownlee IA, Dettmar PW, Pearson JP (2010). Laryngeal mucosa: its susceptibility to damage by acid and pepsin. Laryngoscope.

[14] Campagnolo A, Priston J, Thoen R, Medeiros T, Assunção A (2014). Laryngopharyngeal reflux: diagnosis, treatment, and latest research. Int Arch Otorhinolaryngol.

[15] Im N, Lee DY, Kim B, Kim J, Jung K, Kim TH, Baek S (2019). Role of matrix metalloproteinases 7 in the pathogenesis of laryngopharyngeal reflux: decreased e-cadherin in acid exposed primary human pharyngeal epithelial cells. Int J Mol Sci.

[16] Kim B, Lee H, Im N, Lee DY, Kang CY, Park I, Lee SH, Lee SH, Baek S, Kim TH (2018). Effect of matrix metalloproteinase inhibitor on disrupted E-cadherin after acid exposure in the human nasal epithelium. Laryngoscope.

[17] Altman KW, Haines GR, Hammer ND, Radosevich JA (2003). The H+/K+-ATPase (proton) pump is expressed in human laryngeal submucosal glands. Laryngoscope.

[18] Altman KW, Kinoshita Y, Tan M, Burstein D, Radosevich JA (2011). Western Blot confirmation of the H+/K+-ATPase proton pump in the human larynx and submandibular gland. Otolaryngol Head Neck Surg.

[19] Bao YY, Jiang Q, Li ZW, Yu E, Zhou SH, Yao HT, Fan J, Yong WW (2020). Gastric H(+)/K(+)-ATPase expression in normal laryngeal tissue and laryngeal carcinoma. Onco Targets Ther.

[20] McCormick CA, Samuels TL, Battle MA, Frolkis T, Blumin JH, Bock JM, Wells C, Yan K, Altman KW, Johnston N (2021). H+/K+ATPase expression in the larynx of laryngopharyngeal reflux and laryngeal cancer patients. Laryngoscope.

[21] Kitay AM, Schneebacher M, Schmitt A, Heschl K, Kopic S, Alfadda T, Alsaihati A, Link A, Geibel JP (2018). Modulations in extracellular calcium lead to H+-ATPase-dependent acid secretion: a clarification of PPI failure. Am J Physiol Gastrointest Liver Physiol.

[22] Formánek M, Jančatová D, Komínek P, Matoušek P, Zeleník K (2017). Laryngopharyngeal reflux and herpes simplex virus type 2 are possible risk factors for adult-onset recurrent respiratory papillomatosis (prospective case-control study). Clin Otolaryngol.

[23] Kahrilas PJ, Kia L (2015). Pepsin: a silent biomarker for reflux aspiration or an active player in extra-esophageal mucosal injury?. Chest.

[24] Ali MS, Parikh S, Chater P, Pearson JP (2013). Bile acids in laryngopharyngeal refluxate: Will they enhance or attenuate the action of pepsin?. Laryngoscope.

[25] Galera-Ruiz H, Ríos-Moreno MJ, González-Cámpora R, Ortega I, Fernández A, García-Escudero A, Galera-Davidson H (2012). The cadherin–catenin complex in laryngeal squamous cell carcinoma. Eur Arch Otorhinolaryngol.

[26] Yin C, Zhang S, Zhong J, Zhou S (2020). Pepsin and laryngeal and hypopharyngeal carcinomas. Clin Exp Otorhinolaryngol.

[27] Tan J, Wang L, Mo T, Wang J, Wang M, Li X (2019). Pepsin promotes IL-8 signaling-induced epithelial–mesenchymal transition in laryngeal carcinoma. Cell Int.

[28] Johnston N, Wells CW, Samuels TL, Blumin JH (2010). Rationale for targeting pepsin in the treatment of reflux disease. Ann Otol Rhinol Laryngol.

[29] Johnston N, Dettmar PW, Bishwokarma B, Lively MO, Koufman JA (2007). Activity/stability of human pepsin: implications for reflux attributed laryngeal disease. Laryngoscope.

[30] Kowalik K, Krzeski A (2017). The role of pepsin in the laryngopharyngeal reflux. Otolaryngol Pol.

[31] Johnston N, Dettmar PW, Lively MO, Postma GN, Belafsky PC, Birchall M, Koufman JA (2006). Effect of pepsin on laryngeal stress protein (Sep70, Sep53, and Hsp70) response: role in laryngopharyngeal reflux disease. Aliment Pharmacol Ther.

[32] Choi YS, Na HG, Bae CH, Song SY, Kim YD (2021). Pepsin exposure in a non-acidic environment upregulates mucin 5AC (MUC5AC) expression via matrix metalloproteinase 9 (MMP9)/nuclear factor κB (NF-κB) in human airway epithelial cells. Int Forum Allergy Rh.

[33] Doukas PG, Vageli DP, Sasaki CT, Judson BL (2021). Pepsin promotes activation of epidermal growth factor receptor and downstream oncogenic pathways, at slightly acidic and neutral ph, in exposed hypopharyngeal cells. Int J Mol Sci.

[34] Niu K, Guo C, Teng S, Zhou D, Yu S, Yin W, Wang P, Zhu W, Duan M (2020). Pepsin promotes laryngopharyngeal neoplasia by modulating signaling pathways to induce cell proliferation. PLoS ONE.

[35] Dai Y, Tan J, Deng C, Liu X, Lv Z, Li X (2020). Association of pepsin and DNA damage in laryngopharyngeal reflux-related vocal fold polyps. Am J Otolaryng.

[36] Samuels TL, Zimmermann MT, Zeighami A, Demos W, Southwood JE, Blumin JH, Bock JM, Johnston N (2021). RNA sequencing reveals cancer-associated changes in laryngeal cells exposed to non-acid pepsin. Laryngoscope.

[37] Wang L, Tan J, Wu T, Zhang R, Wu J, Zeng F, Liu Y, Han X, Li Y, Li X (2017). Association between laryngeal pepsin levels and the presence of vocal fold polyps. Otolaryngol Head Neck Surg.

[38] Kim JH, Jang SJ, Yun JW, Jung MH, Woo SH (2018). Effects of pepsin and pepstatin on reflux tonsil hypertrophy in vitro. PLoS ONE.

[39] Kim JH, Jeong H, Kim KM, Lee YJ, Jung MH, Park JJ, Kim JP, Woo SH (2016). Extra-esophageal pepsin from stomach refluxate promoted tonsil hypertrophy. PLoS ONE.

[40] O’Reilly RC, Soundar S, Tonb D, Bolling L, Yoo E, Nadal T, Grindle C, Field E, He Z (2015). The role of gastric pepsin in the inflammatory cascade of pediatric otitis media. JAMA Otolaryngol Head Neck Surg.

[41] Formánek M, Komínek P, Jančatová D, Staníková L, Tomanová R, Vaculová J, Urík M, Šlapák I, Zeleník K (2019). Laryngopharyngeal reflux is a potential risk factor for juvenile-onset recurrent respiratory papillomatosis. Biomed Res Int.

[42] Aldhahrani A, Powell J, Ladak S, Ali M, Ali S, Verdon B, Pearson J, Ward C (2018). The potential role of bile acids in acquired laryngotracheal stenosis. Laryngoscope.

[43] Li T, Chiang JY (2014). Bile acid signaling in metabolic disease and drug therapy. Pharmacol Rev.

[44] Sereg-Bahar M, Jerin A, Hocevar-Boltezar I (2015). Higher levels of total pepsin and bile acids in the saliva as a possible risk factor for early laryngeal cancer. Radiol Oncol.

[45] De Corso E, Baroni S, Salonna G, Marchese M, Graziadio M, Di Cintio G, Paludetti G, Costamagna G, Galli J (2020). Impact of bile acids on the severity of laryngo-pharyngeal reflux. Clin Otolaryngol.

[46] De Corso E, Baroni S, Agostino S, Cammarota G, Mascagna G, Mannocci A, Rigante M, Galli J (2007). Bile acids and total bilirubin detection in saliva of patients submitted to gastric surgery and in particular to subtotal Billroth II resection. Ann Surg.

[47] Sasaki CT, Doukas SG, Costa J, Vageli DP (2019). Biliary reflux as a causal factor in hypopharyngeal carcinoma: New clinical evidence and implications. Cancer.

[48] Vageli DP, Prasad ML, Sasaki CT (2016). Gastro-duodenal fluid induced nuclear factor-κappaB activation and early pre-malignant alterations in murine hypopharyngeal mucosa. Oncotarget.

[49] Sasaki CT, Hajek M, Doukas SG, Vageli DP (2020). The role of bile reflux and its related NF-κB activated pathway in progression of hypopharyngeal squamous cell cancer. Oral Oncol.

[50] Vageli DP, Doukas SG, Sasaki CT (2018). Inhibition of NF-κB prevents the acidic bile-induced oncogenic mRNA phenotype, in human hypopharyngeal cells. Oncotarget.

[51] Sasaki CT, Vageli DP (2016). miR-21, miR-155, miR-192, and miR-375 deregulations related to NF-kappaB activation in gastroduodenal fluid-induced early preneoplastic lesions of laryngeal mucosa in vivo. Neoplasia.

[52] Sasaki CT, Doukas SG, Costa J, Vageli DP (2020). The progressive mutagenic effects of acidic bile refluxate in hypopharyngeal squamous cell carcinogenesis: new insights. Cancers.

[53] Sasaki CT, Doukas SG, Doukas PG, Vageli DP (2021). Weakly acidic bile is a risk factor for hypopharyngeal carcinogenesis evidenced by DNA damage, antiapoptotic function, and premalignant dysplastic lesions in vivo. Cancers.

[54] Wu L, Oshima T, Shan J, Sei H, Tomita T, Ohda Y, Fukui H, Watari J, Miwa H (2015). PAR-2 activation enhances weak acid-induced ATP release through TRPV1 and ASIC sensitization in human esophageal epithelial cells. Am J Physiol Gastrointest Liver Physiol.

[55] Alvarez MMP, Moura GE, Machado MFM, Viana GM, de Souza Costa CA, Tjäderhane L, Nader HB, Tersariol ILS, Nascimento FD (2017). PAR-1 and PAR-2 expression is enhanced in inflamed odontoblast cells. J Dent Res.

[56] Carey RM, Freund JR, Hariri BM, Adappa ND, Palmer JN, Lee RJ (2020). Polarization of protease-activated receptor 2 (PAR-2) signaling is altered during airway epithelial remodeling and deciliation. J Biol Chem.

[57] Xiaopeng B, Tanaka Y, Ihara E, Hirano K, Nakano K, Hirano M, Oda Y, Nakamura K (2017). Trypsin induces biphasic muscle contraction and relaxation via transient receptor potential vanilloid 1 and neurokinin receptors 1/2 in porcine esophageal body. Eur J Pharmacol.

[58] Tanaka Y, Ihara E, Hirano K, Takahashi S, Hirano M, Nakamura K, Akiho H, Oda Y, Takayanagi R (2015). Trypsin-induced biphasic regulation of tone in the porcine lower esophageal sphincter. Eur J Pharmacol.

[59] Shan J, Oshima T, Chen X, Fukui H, Watari J, Miwa H (2012). Trypsin impaired epithelial barrier function and induced IL-8 secretion through basolateral PAR-2: a lesson from a stratified squamous epithelial model. Am J Physiol Gastrointest Liver Physiol.

[60] Cao J, Zhang L, Liu Y, Wang W, Wang Y, Li C, Zhao Y, Li S, Yu L (2020). Properties of a novel animal model of LPR. J Voice.

[61] Silva RO, Bingana RD, Sales TMAL, Moreira RLR, Costa DVS, Sales KMO, Brito GAC, Santos AA, Souza MÂN, Soares PMG (2018). Role of TRPV1 receptor in inflammation and impairment of esophageal mucosal integrity in a murine model of nonerosive reflux disease. Neurogastroenterol Motil.

[62] Suzuki N, Mihara H, Nishizono H, Tominaga M, Sugiyama T (2015). Protease-activated receptor-2 up-regulates transient receptor potential vanilloid 4 function in mouse esophageal keratinocyte. Dig Dis Sci.

[63] Schlueter N, Glatzki J, Klimek J, Ganss C (2012). Erosive-abrasive tissue loss in dentine under simulated bulimic conditions. Arch Oral Biol.

[64] Hurley BP, Jugo RH, Snow RF, Samuels TL, Yonker LM, Mou H, Johnston N, Rosen R (2019). Pepsin triggers neutrophil migration across acid damaged lung epithelium. Sci Rep.

[65] Roh J, Lee Y, Park HT (2006). Effect of acid, pepsin, and bile acid on the stenotic progression of traumatized subglottis. Am J Gastroenterol.

[66] Doukas SG, Cardoso B, Tower JI, Vageli DP, Sasaki CT (2019). Biliary tumorigenic effect on hypopharyngeal cells is significantly enhanced by pH reduction. Cancer Med.

[67] Lillemoe KD, Johnson LF, Harmon JW (1985). Taurodeoxycholate modulates the effects of pepsin and trypsin in experimental esophagitis. Surgery.

[68] Trudgill NJ, Sifrim D, Sweis R, Fullard M, Basu K, McCord M, Booth M, Hayman J, Boeckxstaens G, Johnston BT (2019). British Society of Gastroenterology guidelines for oesophageal manometry and oesophageal reflux monitoring. Gut.

[69] Woodland P, Singendonk M, Ooi J, Nikaki K, Wong T, Lee C, Glasinovic E, Koning R, Lutter R, Benninga MA (2019). Measurement of salivary pepsin to detect gastroesophageal reflux disease is not ready for clinical application. Clin Gastroenterol Hepatol.

[70] Yadlapati R, Adkins C, Jaiyeola D, Lidder AK, Gawron AJ, Tan BK, Shabeeb N, Price CPE, Agrawal N, Ellenbogen M (2016). Abilities of oropharyngeal pH tests and salivary pepsin analysis to discriminate between asymptomatic volunteers and subjects with symptoms of laryngeal irritation. Clin Gastroenterol Hepatol.

[71] Na SY, Kwon OE, Lee YC, Eun YG (2016). Optimal timing of saliva collection to detect pepsin in patients with laryngopharyngeal reflux. Laryngoscope.

[72] Hoppo T, Zaidi AH, Matsui D, Martin SA, Komatsu Y, Lloyd EJ, Kosovec JE, Civitarese AA, Boyd NH, Shetty A (2018). Sep70/Pepsin expression in hypopharynx combined with hypopharyngeal multichannel intraluminal impedance increases diagnostic sensitivity of laryngopharyngeal reflux. Surg Endosc.

[73] Im N, Kim B, Jung K, Baek S (2021). Usefulness of matrix metalloproteinase-7 in saliva as a diagnostic biomarker for laryngopharyngeal reflux disease. Sci Rep.

[74] Katz PO, Gerson LB, Vela MF (2013). Guidelines for the diagnosis and management of gastroesophageal reflux disease. Am J Gastroenterol.

[75] Lechien JR, Mouawad F, Barillari MR, Nacci A, Khoddami SM, Enver N, Raghunandhan SK, Calvo-Henriquez C, Eun Y, Saussez S (2019). Treatment of laryngopharyngeal reflux disease: a systematic review. World J Clin Cases.

[76] Gooi Z, Ishman SL, Bock JM, Blumin JH, Akst LM (2015). Changing patterns in reflux care. Ann Otol Rhinol Laryngol.

[77] Lechien JR, Perazzo PS, Ceccon FP, Eckley CA, Lopes KDC, Maunsell R, Avelino MAG, Akst LM, Sant Anna GD, Imamura R (2020). Management of laryngopharyngeal reflux in Brazil: a national survey. Braz J Otorhinolaryngol.

[78] Lechien JR, Mouawad F, Mortuaire G, Remacle M, Bobin F, Huet K, Nacci A, Barillari MR, Crevier-Buchman L, Hans S (2019). Awareness of european otolaryngologists and general practitioners toward laryngopharyngeal reflux. Ann Otol Rhinol Laryngol.

[79] Park W, Hicks DM, Khandwala F, Richter JE, Abelson TI, Milstein C, Vaezi MF (2005). Laryngopharyngeal reflux: prospective cohort study evaluating optimal dose of proton-pump inhibitor therapy and pretherapy predictors of response. Laryngoscope.

[80] Lechien JR, Saussez S, Muls V, Barillari MR, Chiesa-Estomba CM, Hans S, Karkos PD (2020). Laryngopharyngeal reflux: a state-of-the-art algorithm management for primary care physicians. J Clin Med.

[81] Karkos PD, Wilson JA (2006). Empiric treatment of laryngopharyngeal reflux with proton pump inhibitors: a systematic review. Laryngoscope.

[82] Liu C, Wang H, Liu K (2016). Meta-analysis of the efficacy of proton pump inhibitors for the symptoms of laryngopharyngeal reflux. J Med Biol Res.

[83] Carroll TL, Fedore LW, Aldahlawi MM (2012). pH Impedance and high-resolution manometry in laryngopharyngeal reflux disease high-dose proton pump inhibitor failures. Laryngoscope.

[84] Takeuchi T, Furuta T, Fujiwara Y, Sugimoto M, Kasugai K, Kusano M, Okada H, Suzuki T, Higuchi T, Kagami T (2020). Randomised trial of acid inhibition by vonoprazan 10/20 mg once daily vs rabeprazole 10/20 mg twice daily in healthy Japanese volunteers (SAMURAI pH study). Aliment Pharmacol Ther.

[85] Gyawali CP, Kahrilas PJ, Savarino E, Zerbib F, Mion F, Smout AJPM, Vaezi M, Sifrim D, Fox MR, Vela MF (2018). Modern diagnosis of GERD: the lyon consensus. Gut.

[86] Brisebois S, Merati A, Giliberto JP (2018). Proton pump inhibitors: Review of reported risks and controversies. Laryngoscope Investig Otolaryngol.

[87] Chater PI, Wilcox MD, Brownlee IA, Pearson JP (2015). Alginate as a protease inhibitor in vitro and in a model gut system; selective inhibition of pepsin but not trypsin. Carbohydr Polym.

[88] Wilkie MD, Fraser HM, Raja H (2018). Gaviscon® Advance alone versus co-prescription of Gaviscon® Advance and proton pump inhibitors in the treatment of laryngopharyngeal reflux. Eur Arch Otorhinolaryngol.

[89] Darwish M, Abu EA, Mohammed K (2019). Formulation, optimization, and evaluation of raft-forming formulations containing Nizatidine. Drug Dev Ind Pharm.

[90] Johnston N, Ondrey F, Rosen R, Hurley BP, Gould J, Allen J, DelGaudio J, Altman KW (2016). Airway reflux. Ann N Y Acad Sci.

[91] Kono K, Takahashi A, Sugai H, Umekawa T, Yano T, Kamiyasu K, Teramatsu M, Fujii H (2005). Oral trypsin inhibitor can improve reflux esophagitis after distal gastrectomy concomitant with decreased trypsin activity. Am J Surg.

[92] Vageli DP, Kasle D, Doukas SG, Doukas PG, Sasaki CT (2020). The temporal effects of topical NF-κB inhibition, in the in vivo prevention of bile-related oncogenic mRNA and miRNA phenotypes in murine hypopharyngeal mucosa: a preclinical model. Oncotarget.

[93] Souza RF (2010). Bringing GERD management up to PAR-2. Am J Gastroenterol.

[94] Quilty F, Freeley M, Gargan S, Gilmer J, Long A (2021). Deoxycholic acid induces proinflammatory cytokine production by model oesophageal cells via lipid rafts. J Steroid Biochem.

